# Association between Dietary Share of Ultra-Processed Foods and Urinary Concentrations of Phytoestrogens in the US

**DOI:** 10.3390/nu9030209

**Published:** 2017-02-28

**Authors:** Eurídice Martínez Steele, Carlos A. Monteiro

**Affiliations:** 1 Department of Nutrition, School of Public Health, University of São Paulo, São Paulo 01246-907, Brazil; emar_steele@hotmail.com; 2 Center for Epidemiological Studies in Health and Nutrition, University of São Paulo, São Paulo 01246-907, Brazil

**Keywords:** national health and nutrition examination survey (NHANES), ultra-processed foods, phytoestrogens, lignans, isoflavones, enterolignans

## Abstract

The aim of this study was to examine the relationship between dietary contribution of ultra-processed foods and urinary phytoestrogen concentrations in the US. Participants from cross-sectional 2009–2010 National Health and Nutrition Examination Survey aged 6+ years, selected to measure urinary phytoestrogens and with one 24-h dietary recall were evaluated (2692 participants). Food items were classified according to NOVA (a name, not an acronym), a four-group food classification based on the extent and purpose of industrial food processing. Ultra-processed foods are formulations manufactured using several ingredients and a series of processes (hence “ultra-processed”). Most of their ingredients are lower-cost industrial sources of dietary energy and nutrients, with additives used for the purpose of imitating sensorial qualities of minimally processed foods or of culinary preparations of these foods. Studied phytoestrogens included lignans (enterolactone and enterodiol) and isoflavones (genistein, daidzein, *O*-desmethylangolensin and equol). Gaussian regression was used to compare average urinary phytoestrogen concentrations (normalized by creatinine) across quintiles of energy share of ultra-processed foods. Models incorporated survey sample weights and were adjusted for age, sex, race/ethnicity, family income, and education, among other factors. Adjusted enterodiol geometric means decreased monotonically from 60.6 in the lowest quintile to 35.1 µg/g creatinine in the highest, while adjusted enterolactone geometric means dropped from 281.1 to 200.1 across the same quintiles, respectively. No significant linear trend was observed in the association between these quintiles and isoflavone concentrations. This finding reinforces the existing evidence regarding the negative impact of ultra-processed food consumption on the overall quality of the diet and expands it to include non-nutrients such as lignans.

## 1. Introduction 

Phytoestrogens are the most abundant class of natural xenoestrogens, a group of estrogen-mimicking compounds structurally or functionally related to the human sex hormone 17β-estradiol with the capacity of binding to estrogen receptors [1]. Phytoestrogens may also modulate the concentration of endogenous estrogens by inducing sex hormone binding globulin or through the inhibition of enzymes such as aromatase. In addition to tissue-specific hormonal effects and estrogen receptor-specific effects, phytoestrogens may also exert other biological effects via antioxidant mechanisms [2,3]. In fact, studies have shown that consumption of foods rich in phytoestrogens may protect against diseases and dysfunctions related to aging, mental processes, metabolism, malignant transformation, cardiovascular diseases, breast and prostate cancers, menopausal symptoms, osteoporosis, atherosclerosis and stroke, and neurodegeneration [3,4,5,6]. Yet, more research is needed in order to fully understand the mechanisms of phytoestrogen action. Indeed, even though studies have reported that isoflavone (a type of phytoestrogen) intake has the potential benefit of preventing colon, endometrial and ovarian cancer, the effects on breast cancer risk are more controversial [3]. On the other hand, some authors do not exclude their negative effect on reproductive disorders, even though no adverse events have been reported in humans [3]. Further details on the effects of each type of phytoestrogen have been described elsewhere [1,2,3]. 

Based on their chemical structure and biosynthesis patterns, phytoestrogens have been divided into chalcones, flavonoids (flavones, flavonols, flavanones, isoflavonoids), lignans, stilbenoids, and miscellaneous classes. Lignans and flavonoids are the two main forms [3]. Lignans are polyphenolic components of plant cell walls found in berries, seeds, grains, nuts, fruits, cruciferous vegetables and red wine, with flaxseed being one of the major sources [1]. Flavonoids can be found in berries, wine, grains, nuts, legumes, and especially soybeans and soy-based products which contain relevant amounts of isoflavones genistein and daidzein [1]. 

Dietary phytoestrogens are first metabolized by intestinal bacteria, then absorbed, conjugated in the liver, circulated in plasma and lastly excreted in urine [4]. Gut metabolism is, apparently, key in determining the biological effects of dietary phytoestrogens [2,6]. For example, mammal lignans enterolactone and enterodiol are produced from plant lignans matairesinol, secoisolariciresinol, lariciresinol and pinoresinol, their glycosides, and other precursors in the diet by the microflora in the proximal colon [2]. Bioavailability of isoflavones, on the other hand, depends on the initial hydrolysis of glucose-conjugated isoflavones to corresponding aglycones by colon microbial families [7] to allow the subsequent uptake by enterocytes and the flow through the peripheral circulation [3]. 

During the past decades, analyses of lignan and isoflavone food contents have allowed the compilation of databases to estimate intakes of these compounds, such as the food composition database for isoflavones from The US Department of Agriculture [8,9]. However, accurate measurement intake is limited both because of intake measurement methodology constraints and difficulties with establishing the phytoestrogen content of foods [10]. Furthermore, the lignan or isoflavone concentration within a food varies substantially according to variety, crop season, location and processing methods [11,12,13,14,15]. Also, the fast-growing list of pre-prepared foods, functional foods and dietary supplements available to consumers makes it difficult to have an updated food-intake instrument which fully captures the intake of these phytoestrogens [16]. An alternative approach is estimating human exposure to lignans and isoflavones through the use of biologic samples such as urine [16]. Even though using these types of measurements has its own limitations, several studies have shown that urinary concentrations of phytoestrogens are reliable biomarkers of phytoestrogen intake in both Asian and western populations [16,17,18,19,20,21] and at least one study found a strong correlation between spot urine and serum phytoestrogen concentrations [10].

Ultra-processed foods include sweet or savory snacks, soft drinks, ready meals and other formulations manufactured using several ingredients and a series of processes (hence ‘“ultra-processed”). Most of their ingredients are lower-cost industrial sources of dietary energy. Nutrients and additives are used with the purpose of imitating sensorial qualities of minimally processed foods or of culinary preparations of these foods, or to disguise undesirable sensory qualities of the final product [22,23,24,25,26,27]. Evidence exists that global food supplies are increasingly becoming dominated by these foods [24,27,28,29,30,31] and that consumption of ultra-processed food is associated with excess weight, obesity [32,33,34], and other diet-related non-communicable diseases (NCDs) [35,36]. Also, nationally representative studies carried out in the US [37,38] and other countries [39,40,41,42] have shown that a high dietary contribution of ultra-processed foods renders grossly nutritionally unbalanced diets. Yet, studies carried out to date have focused mainly on nutrients, and did not evaluate the association between ultra-processed food consumption and phytoestrogens in the diet. 

This study aims to expand the knowledge on the impact of ultra-processed food consumption on dietary quality by assessing its relationship with urinary concentrations of phytoestrogens in the US population. 

## 2. Subjects and Methods

### 2.1. Data Source, Population and Sampling

Nationally representative data from the 2009–2010 National Health and Nutrition Examination Survey (NHANES), specifically the dietary component What we eat in America (WWEIA) was utilized. NHANES is a continuous, nationally representative, cross-sectional survey of the non-institutionalized, civilian US residents [43].

The survey included an interview conducted in the home and a subsequent health examination performed at a mobile examination center (MEC), including blood and urine collection. All NHANES examinees were eligible for two 24-h dietary recall interviews. The first dietary recall interview was collected in-person in the MEC [44] while the second was collected by telephone 3–10 days later [45]. Dietary interviews were conducted by trained interviewers using the validated [46,47,48] US Department of Agriculture Automated Multiple-Pass Method [49].

Of the 13,272 people screened in NHANES 2009–2010, 10,537 (79.4%) participated in the household interview and 10,253 (77.3%) also participated in the MEC health examination [50]. A one-third subsample of 2,941 participants 6 years and over (8,591 individuals) was selected to measure urinary phytoestrogens. 

After excluding participants with missing dietary data (129) and an additional 120 with missing urinary phytoestrogens data, 2,692 participants who provided one day of complete dietary intakes were evaluated, 2,411 of which provided two days. The final sample had similar socio-demographic characteristics (gender, age, race/ethnicity, family income and educational attainment) to the full subsample of 2,941 participants selected to measure urinary phytoestrogens (Table S1).

### 2.2. Urinary Phytoestrogen Measurement

Studied phytoestrogens were measured in spot urine samples and included lignans (enterolactone and enterodiol) and isoflavones (genistein, daidzein, *O*-desmethylangolensin, and equol).

Urine specimens were collected the morning after a recommended fast at the MEC, and processed, stored, and shipped to the Division of Laboratory Sciences, National Center for Environmental Health, Centers for Disease Control and Prevention for analysis. Vials were stored under appropriate frozen (–20 °C) conditions until they were shipped to National Center for Environmental Health for testing.

The test principle for the quantitative detection of genistein, daidzein, equol, *O*-desmethylangolensin (*O*-DMA), enterodiol, and enterolactone utilized high performance liquid chromatography–atmospheric pressure photoionization–tandem mass spectrometry (HPLC–APPI–MS/MS). Human urine samples were processed using enzymatic deconjugation of the glucuronidated phytoestrogens followed by size-exclusion filtration. Phytoestrogens were then separated from other urine components by reversed-phase HPLC, detected by APPI–MS/MS, and quantified by isotope dilution. Assay precision was improved by incorporating carbon-13 labeled internal standards for each of the analytes, as well as a 4-methylumbelliferyl glucuronide and 4-methylumbelliferyl sulfate standards to monitor deconjugation efficiency (further details are provided in NHANES Laboratory Procedure Manual) [51]. 

In order to correct for urine dilution, urinary phytoestrogen concentrations were normalized by urinary creatinine (expressed in μg/g creatinine). This was done by dividing each individual’s phytoestrogen concentration value (expressed in ng/mL) by the corresponding urinary creatinine value (expressed in mg/dL). Creatinine was measured using Roche/Hitachi Modular P Chemistry Analyzer (Roche Diagnostics, Indianapolis, IN, USA) at the University of Minnesota [52].

For the sample of 2,692, 12 individuals were below the lower detection limit for enterodiol (0.04 ng/mL), 0 for enterolactone (0.1 ng/mL), 2 for daidzein (0.4 ng/mL), 3 for equol (0.06 ng/mL), 0 for genistein (0.2 ng/mL), and 129 for *O*-desmethylangolensin (0.2 ng/mL) [51]. Several approaches exist to handle left-handed censored data. In NHANES, urinary phytoestrogen measurements below the limits of detection of the used method [51] were replaced with 1/√2 fraction of the detection limit. Treating these left-handed censored values incorrectly may introduce bias when estimating the point and confidence interval of distributions [53]. Still, censoring should not affect estimate reliability in this study, as it has been shown that little bias is introduced by any of the replacement techniques if only a small percentage of the values have been censored (i.e., less than 5%) [54].

### 2.3. Food Classification According to Processing

All recorded food items (*n* = 238,239 Food Codes) were classified according to NOVA (a name, not an acronym), a food classification based on the extent and purpose of industrial food processing [25,55]. NOVA includes four groups: “unprocessed or minimally processed foods” (such as fresh, dry or frozen fruits or vegetables; packaged grains and pulses; grits, flakes or flours made from corn, wheat or cassava; pasta, fresh or dry, made from flours and water; eggs; fresh or frozen meat and fish and fresh or pasteurized milk); “processed culinary ingredients” (including sugar, oils, fats, salt, and other substances extracted from foods and used in kitchens to season and cook unprocessed or minimally processed foods and to make culinary preparations), “processed foods” (including canned foods, sugar-coated dry fruits, salted meat products, cheeses and freshly made unpackaged breads, and other ready-to-consume products manufactured with the addition of salt or sugar or other substances of culinary use to unprocessed or minimally processed foods), and “ultra-processed foods”.

The NOVA group of ultra-processed foods, of particular interest in this study, includes soft drinks, sweet or savory packaged snacks, confectionery and industrialized desserts, mass-produced packaged breads and buns, poultry and fish nuggets and other reconstituted meat products, instant noodles and soups, and many other ready-to-consume formulations of several ingredients. Besides salt, sugar, oils, and fats, these ingredients include food substances not commonly used in culinary preparations, such as modified starches, hydrogenated oils, protein isolates and classes of additives whose purpose is to imitate sensorial qualities of unprocessed or minimally processed foods and their culinary preparations, or to disguise undesirable qualities of the final product. These additives include colorants, flavorings, non-sugar sweeteners, emulsifiers, humectants, sequestrants, and firming, bulking, de-foaming, anti-caking and glazing agents. Unprocessed or minimally-processed foods represent a small proportion of or are even absent from the list of ingredients of ultra-processed products. A detailed definition of each NOVA food group and examples of food items classified in each group has been previously published [37]. The rationale underlying the classification is also shown elsewhere [22,23,24,56,57].

For all food items (Food codes) judged to be a handmade recipe, the classification was applied to the underlying ingredients (Standard Reference codes or SR codes) obtained from the United States Department of Agriculture (USDA) Food and Nutrient Database for Dietary Studies (FNDDS) 5.0 [58] as further explained in a previously published paper [37].

### 2.4. Assessing Energy Content

For this study, Food code energy values as provided by NHANES were used. 

On the other hand, for handmade recipes, the underlying ingredient (SR code) energy values were calculated using variables from both *FNDDS 5.0* [58] and USDA National Nutrient Database for Standard Reference, Release 24 (SR24) [59]. 

### 2.5. Data Analysis

All available day 1 dietary intake data for each participant were utilized. 

Food items were sorted into mutually exclusive food subgroups within unprocessed or minimally processed foods (*n* = 11), processed culinary ingredients (*n* = 4), processed foods (*n* = 4) and ultra-processed foods (*n* = 17), as shown in Table 1. First, the contributions of each of the NOVA food groups and subgroups to total energy intake and across quintiles of the dietary energy contribution of ultra-processed foods (henceforth ‘dietary share of ultra-processed foods’) were evaluated. The group of unprocessed or minimally processed foods was also combined with the group of processed culinary ingredients, as foods belonging to these two groups are usually combined together in culinary preparations and therefore consumed together.

As urinary phytoestrogen concentrations (both in ng/mL and normalized by creatinine) had skewed distributions, these variables were log transformed (using natural logarithms) and geometric means were presented.

The average phytoestrogen urinary concentrations were compared across quintiles of the dietary share of ultra-processed foods using Gaussian regression. Tests of linear trend were performed in order to evaluate the effect of quintiles as a single continuous variable. For each phytoestrogen, four models were explored: (1) crude (in ng/mL); (2) normalized by creatinine (µg/g); (3) normalized by creatinine and adjusted for socio-demographic variables: sex, age group (6–11 years, 12–19 years, 20–39 years, 40–59 years, 60+ years), race/ethnicity (Mexican-American, other Hispanic, non-Hispanic White, non-Hispanic Black, other race including multi-racial), ratio of family income to poverty (categorized based on Supplemental Nutrition Assistance Program (SNAP) eligibility as 0.00–1.30, >1.30–3.50, and >3.50 and above) [43], and educational attainment of respondents for participants aged 20+ years and of household reference person otherwise (<12, 12 years and >12 years); and (4) normalized by creatinine and adjusted for socio-demographic + other variables: socio-demographic variables, difference between recommended and actual energy intake (*z*-score), BMI (body weight divided by height squared, kg/m2: *z*-score for age if <20 years; *z*-score if ≥20 years old), minutes per week of physical activity (*z*-score; estimated separately in <12 and ≥12 years old) and current smoking (yes, no).

As 264 participants had missing values on family income and/or educational attainment, adjusted analysis included 2,428 individuals. Analyses which also adjusted for difference between recommended and actual energy intake, BMI and minutes per week of physical activity included 2,403 individuals.

NHANES survey sample weights were used in all analyses to account for differential probabilities of selection for the individual domains, nonresponse to survey instruments, and differences between the final sample and the total US population. The Taylor series linearization variance approximation procedure was used for variance estimation in all analysis in order to account for the complex sample design and the sample weights [43]. 

Statistical hypotheses were tested using a two-tailed *p* < 0.01 level of significance. Data were analyzed using Stata statistical software package version 12.1 (StataCorp LP, College Station, Texas, USA).

## 3. Results

### 3.1. Contribution of Nova food Groups to Total Energy Intake 

Table 1 presents estimates for the daily energy intake of the US population 6 years and over, the distribution of this intake according to the four NOVA food groups, and the NOVA group distribution across quintiles of the energy share of ultra-processed foods. The population average daily energy intake was 2,153 kcal. Most calories (57.8%) came from ultra-processed foods, 29.2% came from unprocessed or minimally processed foods, 9.8% from processed foods and 3.2% from processed culinary ingredients. An inverse linear trend (*p* < 0.01) was observed in the relationship between the share of ultra-processed foods (quintiles) and the dietary contribution of unprocessed or minimally processed foods, as well as processed culinary ingredients and processed foods. The same applies to individual sub-groups within these three NOVA groups including (but not limited to) lignan and isoflavone food sources such as fruit, grains, roots and tubers, legumes, vegetables, other unprocessed (including nuts and seeds) and other processed (including wine) foods. The energy contribution of most subgroups belonging to ultra-processed foods, many of which are also potential sources of isoflavones [60], increased monotonically from the first to the last quintile of the dietary share of ultra-processed foods, with a few exceptions which showed a slight decrease between the fourth and fifth quintiles (i.e., breakfast cereals or sauces). 

### 3.2. Association between Consumption of Ultra-Processed Foods and Urinary Phytoestrogen Concentrations 

Table 2 presents the average concentrations of urinary phytoestrogens across quintiles of the dietary share of ultra-processed foods. An inverse linear trend (*p* < 0.01) was observed in the association between quintiles of the dietary share of ultra-processed foods and enterolignan concentration averages (geometric mean). Indeed, adjusted enterodiol concentrations decreased monotonically from 60.6 in the lowest quintile to 35.1 µg/g creatinine in the highest quintile, while enterolactone concentrations dropped from 281.1 to 200.1 µg/g creatinine across the same quintiles.

In the association between quintiles of the dietary share of ultra-processed foods and each of the four urinary isoflavone concentrations (genistein, daidzein, *O*-desmethylangolensin, and equol) no significant linear trend was observed. 

## 4. Discussion

In this analysis of US nationally representative data, a strong inverse linear trend was observed in the association between the quintiles of the dietary contribution of ultra-processed foods and urinary concentrations of enterolignans, one of the two main groups of dietary phytoestrogens. Indeed, adjusted enterodiol concentrations decreased monotonically from 60.6 in the lowest quintile to 35.1 µg/g creatinine in the highest quintile, while enterolactone concentrations dropped from 281.1 to 200.1 µg/g creatinine across the same quintiles. This is consistent with the fact that the main dietary sources of lignans are whole foods (berries, seeds, grains, nuts, fruits, and cruciferous vegetables [1]), which are absent from most ultra-processed foods or present in very small amounts.

Studies have found statistically significant correlations between urine and serum concentrations for enterodiol (*r* = 0.83; *r* = 0.62) [10,61] and enterolactone (*r* = 0.94; *r* = 0.84) [10,61], while several intervention studies found that dietary intake of lignan-containing foods leads to increased enterolignan blood concentrations in nearly all individuals [62]. Several studies have also shown that urinary concentrations of enterolignans are reliable biomarkers of lignan intake [16,18,21]. Based on this evidence, we may also conclude that diets rich in ultra-processed foods lead to a reduced lignan bioavailability, either because of low lignan intake, low conversion to enterolignans or low absorption at the colon. Any component altering intestinal flora or its environment, such as diet, may potentially affect the degree to which precursors are converted to enterolignans and absorbed. 

Decreased urinary enterolactone concentrations as observed in high ultra-processed food consumers may have serious health consequences if, as was shown in a study carried out with US NHANES 2003–2010 survey, urinary enterolactone concentrations are inversely associated with serum g-glutamyl transaminase (GGT) levels in adult males and females, with alkaline phosphatase (ALP) in females, and with aspartate aminotransferase (AST) and alanine aminotransferase (ALT) levels in adult males as well [63]. Also, another study carried with US NHANES 2001–2010, found an inverse association between urinary enterolactone levels and obesity, waist circumference, serum triacylglycerols (TAG) levels, fasting glucose levels, fasting insulin levels and metabolic syndrome in adult males, and a direct association with high-density lipoprotein HDL-cholesterol levels [64]. Another study found an inverse association between urinary concentrations of total enterolignans and cardiovascular mortality and between urinary concentrations of both total enterolignans and enterolactone and all-cause mortality [65]. Interestingly, this study also found a direct association between urinary concentration of total isoflavones and daidzein and risk of death from cardiovascular disease and all causes [65].

The somewhat unexpected absence of association between the dietary contribution of ultra-processed foods and the other important group of dietary phytoestrogens—isoflavones—may be explained by the widespread presence of soy in ultra-processed foods [60], in the form of soybean oil, soy flour, soy protein concentrates, soy protein isolates, textured soy protein or soymilk among others [60]. The presence of these ingredients in ultra-processed foods, probably compensate the absence of other food sources of isoflavones in diets rich in ultra-processed foods. 

This study has several strengths. A large, nationally representative sample of the US population was used, increasing generalizability. This investigation was based on individual consumption data, rather than market disappearance or household purchasing data which cannot account for differences between amounts purchased and amounts actually consumed.

Exposure to individual phytoestrogens was assessed by measuring their urinary concentrations, the only option at present to assess dietary intake of lignans as these are not captured by the US Department of Agriculture food composition database (isoflavones are not fully captured either). The advantage of urinary measurements is that they are free of recall bias inherent in self-reported dietary data and represent phytoestrogen intakes from all sources, including those that may be inadequately captured in food composition databases [10,16,65]. An additional advantage of using urinary phytoestrogen testing is that it also captures phytoestrogen metabolites (such as equol and *O*-desmethylangolensin) produced by intestinal bacteria [66].

Potential limitations should be considered. As with most population measures, dietary data obtained by 24-h recalls is imperfect [67]. However, 24-h recalls are the least-biased self-report instrument available. Also, standardized methods and approach of NHANES have been shown to produce accurate intake estimates [46,47,48], and will therefore be suitable for assessing population averages. Previous studies suggest that people with obesity may underreport consumption of foods with caloric sweeteners [68] such as desserts and sweet baked goods [69,70], which may lead to an underestimation of the dietary contribution of ultra-processed foods or a dilution of the association between ultra-processed food consumption and enterodiol and enterolactone urinary concentrations. Although NHANES collects limited information indicative of food processing (i.e., place of meals, product brands), these data are not consistently determined for all food items and may also not provide updated, market-representative nutrient information [71], which could lead to modest over-or underestimation of the dietary contribution of ultra-processed foods. 

A further limitation arises from the use of spot urine samples for the determination of phytoestrogen concentrations as these measurements might be different from those obtained using 24-h urine due to potential circadian rhythm [65]. Even though there have been no studies examining the correlation between spot and 24-h urinary phytoestrogen concentrations, the concentrations of phytoestrogens in spot urine have been reported to be statistically significantly correlated with their concentrations measured in serum [10,61] and with their dietary intake [16,17,18,19,20,21]. Also, even though single measurements in spot urines might not accurately reflect individual usual dietary intake due to within-person variation, in this study we were more interested in the urinary measurements actually reflecting the individual’s dietary intake during the previous 24 h. Taking into account that isoflavone metabolites, like equol, are excreted in the urine within 24 h after exposure [66] and, that peak serum daidzein and genistein concentrations are attained 4–8 h after ingestion while elimination half-lives are in the range of 8–10 h, respectively [72], we may assume this is the case.

## 5. Conclusions

This study shows there is a strong inverse linear trend in the association between the dietary share of ultra-processed foods and urinary concentrations of enterodiol and enterolactone in the US, which probably reflects a lower intake and/or bioavailability of lignans among high consumers of ultra-processed foods. This finding reinforces the existing evidence regarding the negative impact of ultra-processed food consumption on the overall quality of the diet [37,38,39,40,41,42], and expands it to include non-nutrients such as lignans.

## Figures and Tables

**Table 1:** Distribution (%) of the total daily per capita energy intake (kcal) according to NOVA food groups by quintiles of the dietary share of ultra-processed foods ^a^.

		Quintile of Dietary Share of Ultra-Processed Foods (% of Total Energy Intake) ^b^				
	All Quintiles	Q1	Q2	Q3	Q4	Q5
	(*n* = 2,692)	(*n* = 539)	(*n* = 530)	(*n* = 521)	(*n* = 540)	(*n* = 562)
	(2,153 kcal)	(2,040.5 kcal)	(2,212.1 kcal)	(2,143.0 kcal)	(2,143.9 kcal)	(2,227.6 kcal)
**Unprocessed or minimally processed foods**	***29.2***	***50.7***	***35.7***	***29.5***	***20.8***	*9.4* *
Meat (includes poultry)	8.2	13.2	10.5	8.7	6.3	2.4 *
Fruit and freshly squeezed fruit juices	4.7	7.7	5.1	5.2	3.6	2.0 *
Milk and plain yoghurt	4.3	5.6	4.6	5	4.1	2.2 *
Grains	3	7.4	3.9	1.9	1.4	0.4 *
Roots and tubers	1.4	2.3	1.9	1.8	0.9	0.4 *
Eggs	1.5	2.1	2.1	1.7	1.3	0.5 *
Pasta	1.3	2.8	1.8	0.9	0.9	0.2 *
Fish and sea food	1	1.9	1.3	0.9	0.5	0.4 *
Legumes	0.9	2	1.1	0.7	0.3	0.1 *
Vegetables	0.7	1.3	0.7	0.7	0.5	0.3 *
Other unprocessed or minimally processed foods ^1^	2	4.4	2.5	1.9	0.9	0.4 *
**Processed culinary ingredients**	***3.2***	***5.6***	***4.1***	***3.1***	***2.1***	*1.0* *
Sugar ^2^	1.3	1.9	1.7	1.5	0.9	0.5 *
Plant oils	1.3	2.7	1.7	0.9	0.7	0.3 *
Animal fats ^3^	0.5	0.7	0.6	0.6	0.5	0.2 *
Other processed culinary ingredients ^4^	0.04	0.12	0.04	0.03	0.01	0.01
**Unprocessed or minimally processed foods + Processed culinary ingredients**	***32.4***	***56.2***	***39.8***	***32.6***	***22.9***	*10.4* *
**Processed foods**	***9.8***	***15.3***	***13.2***	***9.2***	***7.5***	*3.9* *
Cheese	3.5	4	4.6	3.8	3.3	2.0 *
Ham and other salted, smoked or canned meat or fish	1.3	1.4	1.6	1.7	1.5	0.6
Vegetables and other plant foods preserved in brine	0.8	0.8	0.9	0.7	0.7	0.3 *
Other processed foods ^5^	4.2	9.1	6.1	2.9	2.1	0.9 *
**Ultra-processed foods**	***57.8***	***28.5***	***47***	***58.2***	***69.6***	*85.6* *
Breads	9.8	6.9	9.8	11.5	11.5	9.4 *
Soft and fruit drinks ^6^	7.3	3.1	5.3	7	8.9	11.9 *
Cakes, cookies and pies	5.7	2	4.3	6.7	7.7	7.6 *
Salty-snacks	4.5	1.6	3.9	4.2	5.5	7.4 *
Frozen and shelf-stable plate meals	3.6	0.6	2.1	2.6	4.6	7.9 *
Pizza (ready-to-eat/heat)	3.7	0.2	1.5	2.7	4.6	9.8 *
Breakfast cereals	2.5	1.7	2.6	2.9	2.8	2.7
Sauces, dressings and gravies	2.5	2	2.4	2.8	3.4	1.9
Reconstituted meat or fish products	2.5	0.6	2.6	2.3	3.1	3.9 *
Sweet-snacks	2.4	1.3	1.9	2.1	3.1	3.8 *
Ice cream and ice pops	2.1	0.8	1.4	2.1	2.6	3.7 *
Desserts ^7^	1.7	1.5	1.4	1.6	1.9	1.9
French fries and other potato products	1.7	0.4	0.9	1.7	2	3.6 *
Sandwiches and hamburgers on bun (ready-to-eat/heat)	1.5	0.1	0.6	0.9	1.7	3.9 *
Milk-based drinks	1.4	0.8	1.3	1.3	1.4	2
Instant and canned soups	0.8	0.7	0.5	1	0.9	0.9
Other ultra-processed foods ^8^	3.9	4	4.4	4.6	3.7	2.9
**Total**	**100**	**100**	**100**	**100**	**100**	**100**

^a^ Subsample of US population aged 6+ years (National Health and Nutrition Examination Survey, NHANES 2009–2010); ^b^ Mean (range) dietary share of ultra-processed foods per quintile: first = 28.5 (1.6–39.5); second = 47.0 (39.5–52.9); third = 58.2 (52.9–63.5); fourth = 69.6 (63.5–75.9); fifth = 85.6 (76.0–100); ^1^ Including nuts and seeds (unsalted); yeast; dried fruits (without added sugars) and vegetables; non pre-sweetened, non-whitened, non-flavored coffee and tea; coconut water and meat; homemade soup and sauces; flours; tapioca; ^2^ Including honey, molasses, maple syrup (100%); ^3^ Including butter, lard and cream; ^4^ Including starches; coconut and milk cream; unsweetened baking chocolate, cocoa powder and gelatin powder; vinegar; baking powder and baking soda; ^5^ Including salted or sugared nuts and seeds; peanut, sesame, cashew and almond butter or spread; beer and wine; ^6^ Including energy drinks, sports drinks, nonalcoholic wine; ^7^ Including ready-to-eat and dry-mix desserts such as pudding; ^8^ Including soy products such as meatless patties and fish sticks; baby food and baby formula; dips, spreads, mustard and catsup; margarine; sugar substitutes, sweeteners and all syrups (excluding 100% maple syrup); distilled alcoholic drinks; * Significant linear trend across all quintiles (*p* < 0.01), both in unadjusted and models adjusted for sex, age group (6–11, 12–19, 20–39, 40–59, 60+ years), race/ethnicity (Mexican-American, other Hispanic, non-Hispanic White, non-Hispanic Black and other race—including multi-racial) ratio of family income to poverty (Supplemental Nutrition Assistance Program, SNAP 0.00–1.30, >1.30–3.50, and >3.50 and over) and educational attainment (<12, 12 years and >12 years).

**Table 2:** Phyto-estrogen concentrations according to the quintiles of the dietary share of ultra-processed foods ^a^.

			Quintile of Dietary Share of Ultra-Processed Foods (% of Total Energy Intake) ^b^					
			Q1	Q2	Q3	Q4	Q5	All Quintiles
Enterolignans (GM ^c^)	Enterodiol	Crude (ng/mL) (*n* = 2,692)	52.7	42.9	38.4	35.8	33.1 *	40.05
		Normalized by creatinine (µg/g) (*n* = 2,692)	61.2	49.2	39.8	38.3	31.6 *	
		Normalized and adjusted for socio-demographic variables ^d^ (*n* = 2,428)	60.8	51.9	38.3	39.3	33.6 *	
		Normalized and adjusted for socio-demographic + other variables (*n* = 2,403) ^e^	60.6	50.7	38.5	40.0	35.1 *	
	Enterolactone	Crude (ng/mL)	255.6	224.4	226.2	209.7	176.4	216.9
		Normalized by creatinine (µg/g)	297.1	257.2	234.4	224.3	168.4 *	
		Normalized and adjusted for socio-demographic variables ^d^	291.8	261.2	219.0	237.5	186.9 *	
		Normalized and adjusted for socio-demographic + other variables ^e^	281.1	258.0	222.8	245.1	200.1	
Isoflavones (GM)	Daidzein	Crude (ng/mL)	57.3	66.8	70.9	70.0	82.3	69.0
		Normalized by creatinine (µg/g)	66.6	76.6	73.5	74.9	78.6	
		Normalized and adjusted for socio-demographic variables ^d^	67.7	79.9	72.1	74.3	71.7	
		Normalized and adjusted for socio-demographic + other variables ^e^	68.9	79.8	72.5	74.9	71.6	
	*O*-Desmethylangolensin (*O*-DMA)	Crude (ng/mL)	4.2	4.9	4.3	4.9	5.5	4.7
		Normalized by creatinine (µg/g)	4.9	5.6	4.4	5.2	5.2	
		Normalized and adjusted for socio-demographic variables ^d^	5.0	5.8	4.3	5.2	5.1	
		Normalized and adjusted for socio-demographic + other variables ^e^	5.1	5.7	4.2	5.3	5.2	
	Equol	Crude (ng/mL)	6.8	7.6	8.5	7.8	9.0	7.9
		Normalized by creatinine (µg/g)	7.9	8.7	8.8	8.4	8.6	
		Normalized and adjusted for socio-demographic variables ^d^	8.9	8.9	8.7	8.2	7.9	
		Normalized and adjusted for socio-demographic + other variables ^e^	8.8	8.9	8.8	8.2	7.9	
	Genistein	Crude (ng/mL)	27.9	29.4	35.6	31.5	38.8	32.4
		Normalized by creatinine (µg/g)	32.5	33.7	36.9	33.7	37.1	
		Normalized and adjusted for socio-demographic variables ^d^	32.4	34.8	35.9	32.7	34.6	
		Normalized and adjusted for socio-demographic + other variables ^e^	32.6	34.8	36.4	32.8	34.4	

^a^ Subsample of US population aged 6+ years (NHANES 2009–2010); ^b^ Mean (range) dietary share of ultra-processed foods per quintile: first = 28.5 (1.6–39.5); second = 47.0 (39.5–52.9); third = 58.2 (52.9–63.5); fourth = 69.6 (63.5–75.9); fifth = 85.6 (76.0–100); ^c^ GM = geometric mean; ^d^ Normalized by creatinine (µg/g) and adjusted for sex, age group, race/ethnicity, ratio of family income to poverty and educational attainment; ^e^ Normalized by creatinine (µg/g) and adjusted for all socio-demographic variables + difference between recommended and actual energy intake (*z*-score), BMI (body weight divided by height squared, kg/m2: *z*-score), minutes per week of physical activity (*z*-score) and current smoking; * Significant linear trend across all quintiles (*p* < 0.01).

## Supplementary Materials

The following are available online at http://www.mdpi.com/2072-6643/9/3/209/s1, Table S1. Characteristics of study participants and full subsample of participants selected to measure urinary phytoestrogens. Subsample of US population aged 6+ years (NHANES 2009–2010).
