# Supplementary material for: Association between Dietary Share of Ultra-Processed Foods and Urinary Concentrations of Phytoestrogens in the US
Source: Nutrients. 2017 Feb 28;9(3):209. doi: 10.3390/nu9030209 (PMC5372872; doi:10.3390/nu9030209)
Supplement: Supplementary file 1 [file nutrients-09-00209-s001.docx]

Supplementary Materials: Association between Dietary Share of Ultra-Processed Foods and Urinary Concentrations of Phytoestrogens in the US

Eurídice Martínez Steele and Carlos A. Monteiro

**Table S1.** Characteristics of study participants and full subsample of participants selected to measure urinary phytoestrogens. Subsample of US population aged 6+ years (NHANES 2009–2010).

|  |  | **Full Subsample (*n* = 2941)** | **Study Sample (*n* = 2692)** |
| --- | --- | --- | --- |
| Gender | Men | 48.8 | 49.1 |
|  | Women | 51.2 | 50.9 |
| Age | 6 to 11 | 8.8 | 8.5 |
|  | 12 to 19 | 12.0 | 12.4 |
|  | 20 to 39 | 29.3 | 29.8 |
|  | 40 to 59 | 30.3 | 29.6 |
|  | 60 and over | 19.6 | 19.7 |
| Race/ethnicity | Mexican American | 9.9 | 10.3 |
|  | Other Hispanic | 5.1 | 5.2 |
|  | Non-Hispanic White | 65.0 | 64.8 |
|  | Non-Hispanic Black | 11.9 | 12.0 |
|  | Other Race (including Multi-Racial) | 8.0 | 7.7 |
| Income to poverty ^a^ | 0.00–1.30 | 22.9 | 23.2 |
|  | >1.30–3.50 | 38.2 | 37.8 |
|  | >3.50 and above | 38.8 | 39.1 |
| Educational attainment ^b^ | <12 years | 19.1 | 19.0 |
|  | 12 years | 22.9 | 23.2 |
|  | >12 years | 58.0 | 57.7 |

^a^ Full subsample: 269 with missing income values; study sample: 241 with missing income values; ^b^ Full subsample: 36 with missing education values; study sample: 32 missing education values.
